# KD_ConvNeXt: knowledge distillation-based image classification of lung tumor surgical specimen sections

**DOI:** 10.3389/fgene.2023.1254435

**Published:** 2023-09-18

**Authors:** Zhaoliang Zheng, Henian Yao, Chengchuang Lin, Kaixin Huang, Luoxuan Chen, Ziling Shao, Haiyu Zhou, Gansen Zhao

**Affiliations:** ^1^ South China Normal University, Guangzhou, China; ^2^ Key Lab on Cloud Security and Assessment Technology of Guangzhou, Guangzhou, China; ^3^ SCNU & VeChina Joint Lab on BlockChain Technology and Application, Guangzhou, China; ^4^ The First School of Clinical Medicine, Guangdong Medical University, Zhanjiang, China; ^5^ Department of Thoracic Surgery, Guangdong Provincial People’s Hospital, Guangdong Academy of Medical Sciences, Guangzhou, China; ^6^ Jinan University-University of Birmingham Joint Institute at Jinan University, Guangdong, China

**Keywords:** lung cancer classification, knowledge distillation, Swin Transformer, ConvNeXt, lung tumor surgical specimen sections

## Abstract

**Introduction:** Lung cancer is currently among the most prevalent and lethal cancers in the world in terms of incidence and fatality rates. In clinical practice, identifying the specific subtypes of lung cancer is essential in diagnosing and treating lung lesions.

**Methods:** This paper aims to collect histopathological section images of lung tumor surgical specimens to construct a clinical dataset for researching and addressing the classification problem of specific subtypes of lung tumors. Our method proposes a teacher-student network architecture based on a knowledge distillation mechanism for the specific subtype classification of lung tumor histopathological section images to assist clinical applications, namely KD_ConvNeXt. The proposed approach enables the student network (ConvNeXt) to extract knowledge from the intermediate feature layers of the teacher network (Swin Transformer), improving the feature extraction and fitting capabilities of ConvNeXt. Meanwhile, Swin Transformer provides soft labels containing information about the distribution of images in various categories, making the model focused more on the information carried by types with smaller sample sizes while training.

**Results:** This work has designed many experiments on a clinical lung tumor image dataset, and the KD_ConvNeXt achieved a superior classification accuracy of 85.64% and an F1-score of 0.7717 compared with other advanced image classification methods

## 1 Introduction

The lung is a vital organ of the human body and is responsible for the respiratory and metabolic functions of the body. In recent years, lung cancer has been ranked as the leading cause of cancer-related deaths worldwide, accounting for more than a quarter (26%) of all cancers (Viale, 2020). The exact type of lung tumor pathology is also an essential factor in the next step of the surgical process.

**FIGURE 1:**
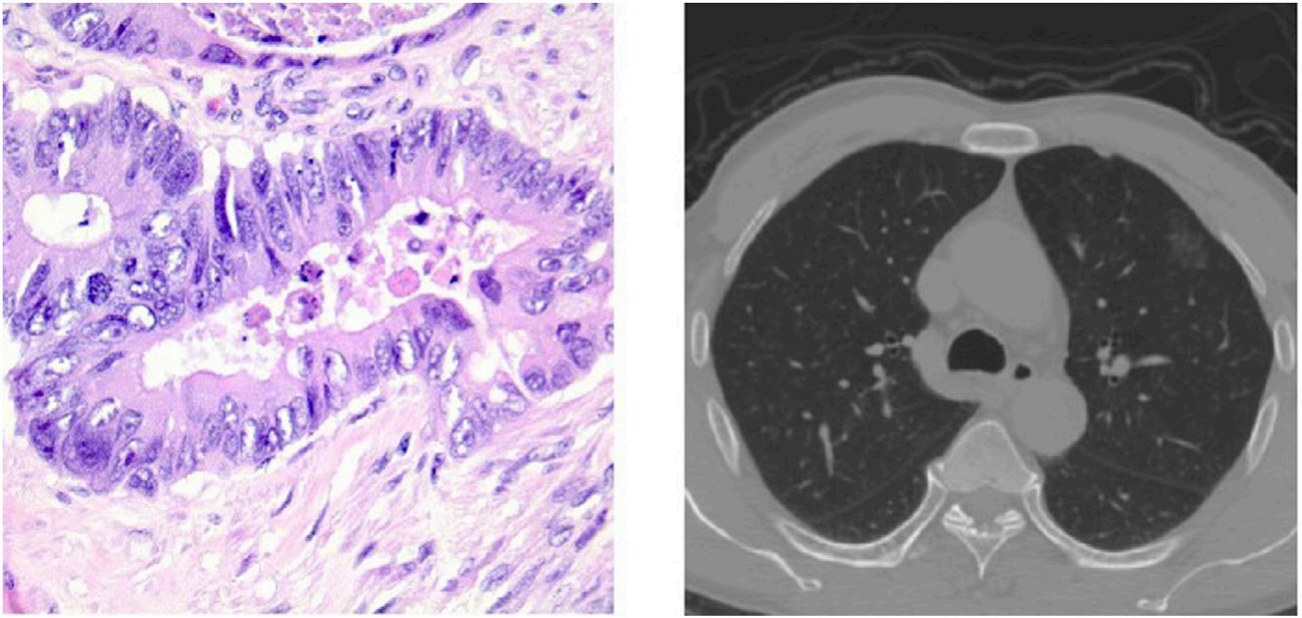
Pathological classification of lung neoplasms: a histopathological slice of lung tumor and CT images of the chest.

Currently, various pathological classifications of lung tumors mainly rely on intraoperative freezing, postoperative paraffin section or puncture biopsy specimen production staining observation, and subsequent immunohistochemical analysis, among which the gold standard for confirming the diagnosis is the paraffin pathology results. However, more accurate pathological typing can be obtained by analyzing pathological sections, as shown in Figure 1, which takes a long time in the scale of days and does not allow immediate determination of the pathological type at the time of obtaining the specimen. Computed tomography (CT) is also a commonly used method for diagnosing lung lesions, which can reflect information on the shape, number, and location of lung nodules and has significant clinical value (Han et al., 2021). Some experienced clinicians can roughly determine the benign and malignant degree of lung tumors by observing the CT images of the chest as shown in Figure 1, but the accuracy is overly dependent on subjective factors such as empirical judgment and has a high rate of misdiagnosis.

Deep learning techniques have recently made a breakthrough in various computer vision tasks with exciting results. In particular, convolutional neural network (CNN)-based image classification algorithms have succeeded dramatically in classification tasks for imaging such as CT, MRI, and pathology images (Yang et al., 2017; Sun et al., 2020). Some studies have shown that deep learning can identify skin cancers in dermoscopic images and determine gastric tumor staging in gastroscopic embodiments with relatively good accuracy compared to pathology results (Esteva et al., 2017; Cho et al., 2019). Other studies based on deep learning techniques have been published in cervical cancer, oral cancer, and bladder cancer (Hu et al., 2019; Shkolyar et al., 2019; Camalan et al., 2021).

There are two main technical routes based on existing deep-learning techniques for lung cancer diagnosis and classification:

1. Lung cancer classification methods based on low-dose computed tomography (CT) images (Anderson and Davis, 2018), which are mainly studied based on public datasets such as LIDC-IDRI and LUNA16 (Gugulothu and Balaji, 2023; Liu et al., 2023; Zhu et al., 2023).
2. Deep learning classification algorithms based on histopathological images, which are mainly studied based on the LC25000 dataset (Masud et al., 2021), digital full-slide images (WSIs) and TCGA34, and other datasets combined with deep learning classification algorithms for the pathological classification of lung tumors (Halder and Dey, 2023; Jeyaraj and Nadar, 2023; Omar et al., 2023).

Despite some progress made by works (Gugulothu and Balaji, 2023; Halder and Dey, 2023; Jeyaraj and Nadar, 2023; Liu et al., 2023; Omar et al., 2023; Zhu et al., 2023) in the task of diagnostic classification of lung cancer, the pathological classification of lung tumors currently faces the following challenges:

• The first is either only preliminarily screening lung tumors for benign and malignant degrees or mixing histopathological images of lung cancer and other cancers to differentiate and classify them, rather than accurately classifying specific subtypes of lung cancer.
• Existing methods fail to take full advantage of the vast amount of other data available in modern clinics, and using routinely obtained images of surgical specimen sections for histological classification may be necessary for diagnostic and therapeutic decisions, as innovative tools for clinical data evaluation are needed to augment biopsies and help better characterize the disease, given the complexity of lung cancer classification and the limitations of current practice.
• The surgical management of lung cancer requires intraoperative frozen pathology analysis of the tumor, during which the patient waits on the operating table for at least 30 min. There are two hot issues of concern: how to reduce the patient’s waiting time on the operating table to reduce the risk of surgery and identifying specific subtypes of lung tumors more accurately and efficiently.

This work aims to collect raw images of lung tumor surgical specimen sections, create a clinical dataset image by cropping the features thoroughly examined by doctors as ROI regions, and investigate the efficacy of deep learning methods for quick classification on this dataset. The goal is to give doctors timely references for surgical strategies and to increase their productivity and diagnostic and treatment decision accuracy. Our method uses the ConvNeXt (Liu et al., 2022) as a student network and the Swin Transformer model (Liu et al., 2021) as a teacher model for knowledge distillation in the intermediate feature mapping layer, and the softmax output layer (Hinton et al., 2015) is applied to solve the problems of lung cancer images classification. Also, to effectively improve the model’s classification performance, we processed the dataset with a super-resolution denoising algorithm. This work performed data enhancement to better learn the features of lung tumors. Overall, the contributions of this paper can be summarized as follows:

• This work constructed a dataset of 2,221 raw images of the ROI regions of lung tumors. Due to the irregularities in processing images in hospitals, we used the Real-ESRGAN (Wang et al., 2021) algorithm to super-resolution process and denoise them to solve the problems of low resolution and noise in the clinically processed dataset, which can improve the accuracy of lung cancer-specific subtype classification. In addition, this work has explored a new and unprecedented route to rapidly predict specific subtypes of liver tumors, which can assist physicians in roughly deciding on subsequent surgical steps and treatment strategies while waiting for the results of intraoperative frozen pathology analysis.
• Our method proposed a teacher–student structure based on the knowledge distillation mechanism called KD_ConvNeXt, which can effectively improve the student network’s feature extraction and fitting ability. Also, the training of the student network will be more biased towards the categories with a smaller sample size, improving the classification ability of clinical datasets with a severe imbalance of category data. The model’s accuracy is not significantly different from existing deep learning techniques based on pathology slides and CT images and is of great clinical reference.
• The proposed approach conducted many comparative experiments and experimental ablation analyses as well as selected advanced image classification methods for comparison to demonstrate the validity and advancement of the proposed framework in classifying specific pathological subtypes of lung tumors and to analyze the existence of shortcomings and deficiencies.

The rest of this paper is organized as follows. In Section 2, this paper first reviewed the related work on the pathological classification of lung cancer. This paper detailed the clinical dataset and the detailed structure of the model in Section 3. We describe the starting point of our experimental design and experimental implementation details in Section 4. Then, we compared the proposed method with some advanced image classification methods, including the comparison of evaluation metrics for image multiclassification and the analysis of ablation experiments in Section 5. Finally, we conclude our work in the Section 6.

## 2 Related work

Currently, studies (Gugulothu and Balaji, 2023; Halder and Dey, 2023; Jeyaraj and Nadar, 2023; Liu et al., 2023; Omar et al., 2023; Zhu et al., 2023) have made some progress in the task of lung tumor pathology classification. These methods include two main technical lines: lung cancer classification methods based on CT images (Gugulothu and Balaji, 2023; Liu et al., 2023; Zhu et al., 2023) and deep learning classification algorithms based on histopathological images (Halder and Dey, 2023; Jeyaraj and Nadar, 2023; Omar et al., 2023).

### 2.1 Lung cancer classification methods based on CT images

Zhu et al., (2023) proposed the DEPMSCNet model, which has high sensitivity and a low false-positive rate for detecting lung nodules. In the feature extraction stage, the model uses REPSA-MSC to extract multi-scale information from the feature maps, while introducing adaptive convolutional branching to detect contextual information at each location of the multi-scale. Secondly, the DSAM (Dual Path Spatial Attention Module) proposed in this model acquires sensory field information from two branches, combining low-level feature map information with high-level semantic information. The model has been evaluated on the public Lung Nodule Analysis (LUNA16) challenge dataset with a sensitivity of 0.988 and a Competitive Performance Measure (CPM) of 0.963.

Liu et al., (2023) proposed a novel asymmetric residual network called 3D-ARCNN that utilizes 3D features and spatial information of lung nodules to improve classification performance. The framework employs an internal cascaded multilevel residual model for fine-grained learning and multilayer asymmetric convolution of lung nodule features to address the problem of significant neural network parameters and poor reproducibility. Through experiments on the publicly available LUNA16 image dataset, the detection sensitivity was found to be 95.8%, and the average CPM index is 0.912.

Gugulothu and Balaji, (2023) first preprocessed the input lung images to remove non-informatics blocks using step deviation mean multilevel thresholding (SDMMT). Afterward, the LN portion is detected using the earliest event network classifier, and essential features are selected using the Horse-Drop Optimization Algorithm (MD-HHOA). The study utilized the publicly accessible Lung Image Database Consortium Image Set (LIDC-IDRI) dataset, and the experimental results show that the proposed method has an accuracy of 97.11%, sensitivity of 96.98%, and specificity of 94.34% for detecting nodules, respectively.

### 2.2 Deep learning classification algorithms based on histopathological images

Halder and Dey, (2023) proposed a novel deep learning framework based on image morphology for lung cancer subtype classification. The framework combines morphology-based pathways with attention blocks that can accurately and efficiently capture morphological variants of lung cancer subtypes and deep features extracted from convolutional and morphological ways for lung cancer subtype classification. This study analyzed the performance of the proposed framework on a publicly available dataset and achieved a sensitivity, specificity, average accuracy, precision, and F1-score of 98.33%, 97.76%, 98.96%, 99.12%, and 98.72%, respectively.

Omar et al., (2023) proposed an integrated migration learning model for fast lung and colon cancer diagnosis by utilizing multiple migration learning models and integrating them for better performance. The accuracies of MobileNet V1, Inception V3, and VGG16 for lung and colon cancer detection are 98.32%, 98%, and 96.93%, respectively, whereas the integrated model of the study has an accuracy of 99.44%. The results of this study indicate that the proposed method is superior to existing models and can therefore be used in clinics to assist medical staff in detecting lung and colon cancers.

Jeyaraj and Nadar, (2023) compared the proposed LDCNN with AlexNet and EfficientNet on benchmark datasets such as MS-COCO, LC25000, and multi-class Kather datasets. The empirical experimental performance metrics obtained by the proposed LDCNN in this study outperform the baseline convolutional neural network architecture, achieving 99.6% accuracy, 98.4% sensitivity, 97.9% specificity, and a 99.1% F1-score. The lightweight feature-specific learning network proposed in this study thus achieved steady improvements in medical annotation work and classification.

### 2.3 Knowledge distillation

Knowledge distillation has emerged as a prominent model compression technique, garnering significant attention in deep learning. It involves a teacher–student training framework, where a trained teacher model imparts knowledge to a student model through a process known as distillation (Hinton et al., 2015). This allows the transfer of knowledge from a complex teacher model to a simpler student model, albeit with a minor sacrifice in performance. Various forms of knowledge can be utilized, such as output feature knowledge, intermediate feature knowledge, relational feature knowledge, and structural feature knowledge. Output feature knowledge primarily encompasses the last layer features of the teacher model, incorporating insights into logical units and soft targets (Zhang et al., 2022; Xu et al., 2023). It enables students to learn the teacher’s final predictions, aiming to achieve a similar predictive performance. Knowledge distillation of intermediate features involves extracting hints from the middle layer of the teacher model, guiding the output of the student model’s intermediate layer (Okamoto et al., 2022; Zhao et al., 2022). This approach utilizes not only the output feature knowledge of the teacher model but also the implicit layer’s feature map knowledge. Relational feature knowledge focuses on capturing relationships between different layers of the teacher model and other data samples (He et al., 2022; Shen and Xing, 2022; Zhang et al., 2023). It aims to establish a consistent relational mapping, facilitating the student model’s enhanced understanding of relational knowledge from the teacher model.

## 3 Methods

In this section, our method first details the collection requirements and the construction process of the clinical dataset, then introduces the teacher–student structure consisting of ConvNeXt and Swin Transformer, and finally presents the loss function of the proposed method in this paper.

### 3.1 Datasets description

The natural images of the lung tumor’s surgical specimen section are obtained by dissecting the tumor in half, fully exposing the tumor section, and then taking more than three to five images from two to three different angles. The specific subtype classification of lung tumors is obtained by refining the three pathological subtypes of lung cancer, adenocarcinoma *in situ* (AIS), micro-invasive adenocarcinoma (MIA), and invasive adenocarcinoma (IA) again because the pathological histological subtypes of IA can be divided into highly differentiated adenocarcinoma, moderately differentiated adenocarcinoma, and poorly differentiated adenocarcinoma, which are also called Grade 1, Grade 2, and Grade 3. These three histological subtypes can predict the prognosis of patients after surgery, and studies showed that AIS and MIA have better predictions than Grade 1, Grade 2, and Grade 3.

**FIGURE 2:**
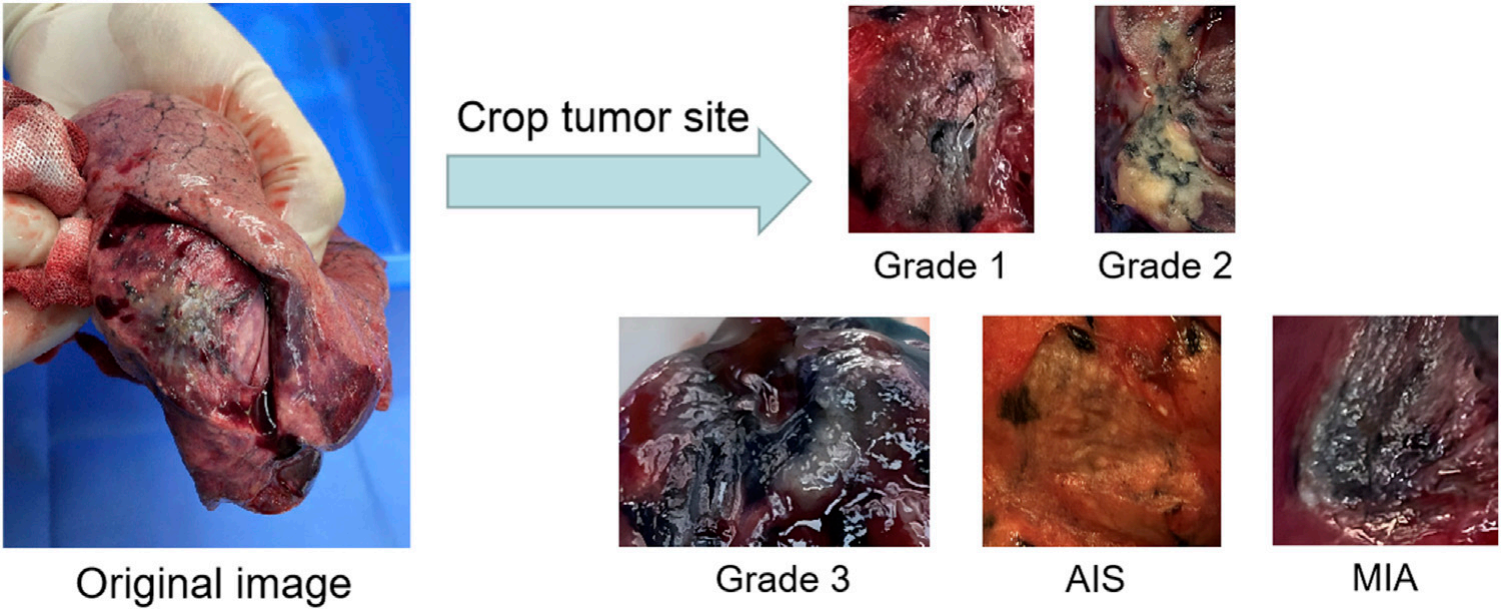
The process of building the clinical dataset of lung tumor: the images obtained by cropping the ROI regions of the tumor section are classified into Grade 1, Grade 2, Grade 3, AIS and MIA.

**FIGURE 3:**
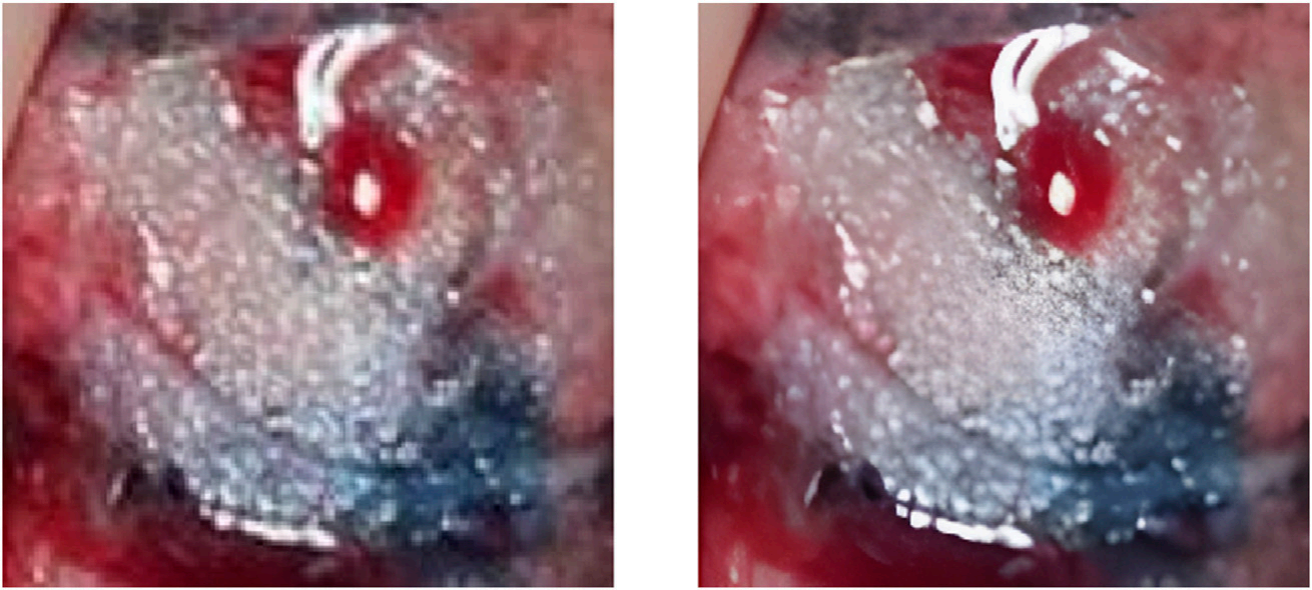
The left image is the original ROI image, and the right image is the image after processing by the Real-ESRGAN algorithm.

To evaluate the practical effects of the model in clinical applications, clinical lung tumor image data were obtained from Guangdong Provincial People’s Hospital, which consisted of 1,245 lesions with 2,221 images in total. Generally speaking, the images obtained from clinical photography may contain some backgrounds irrelevant to cancer analysis. Removing these non-informative regions can significantly reduce the computational cost and ensure the validity of the training samples, so each lesion image is the image obtained by cropping the tumor site after removing some irrelevant backgrounds such as normal lung tissues from the original image, illustrated by Figure 2. Also, the clinical image dataset taken in the field undergoes various processes, such as camera blurring and image compression when cropping the ROI region, which makes the images blurred and degraded. To improve the resolution of the images and remove the noise, the Real-ESRGAN algorithm (Wang et al., 2021) is used to process the clinical image dataset, illustrated by Figure 3.

### 3.2 Model design

Enhancing the feature extraction component to ensure the accuracy of pathological classification in the lung tumor clinical dataset is essential to achieving multi-scalability and accuracy of the retrieved features. Therefore, this work chose a teacher–student architecture of knowledge distillation to improve the model accuracy and realize the domain migration between image labels. In the teacher–student architecture, the teacher network provides supervision information, and the student network is responsible for learning. For the medical image classification task, if the entropy of soft targets is higher than that of hard targets, it is evident that the student will learn more information, which improves the classification accuracy and the generalization ability of the student network. The retrieved features exhibit a multi-scale notion due to the hierarchical feature extraction facilitated by Swin Transformer, which can be divided into multiple Blocks. The shifting operation enables interaction between neighboring windows, thereby flexibly providing information at different scales. The properties of shifting are learned through moving windows, resulting in self-attentiveness computed within the window. To enhance the capacity of the convolutional neural network for feature extraction and classification, Swin Transformer is employed as the teacher model for knowledge distillation in the ConvNets network. The student network, ConvNeXt, is specifically chosen as it is a framework and training method derived from Vision Transformer that enhances the initial ResNet50 model. This decision is primarily based on the comparable model architectures of Swin Transformer and ConvNeXt, allowing for easy separation into matching layer blocks for the independent distillation of feature layers.

**FIGURE 4:**
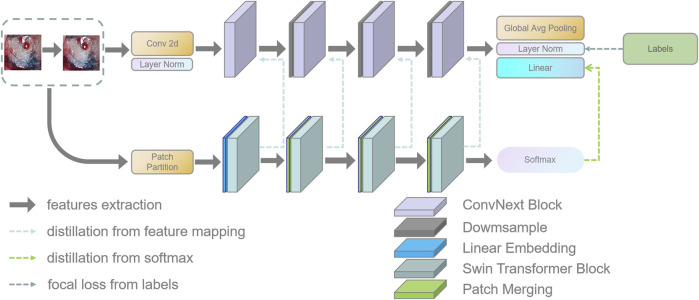
This figure shows the details of the teacher–student network architecture. (1) ConvNeXt and Swin Transformer are divided into four parts according to their structures. (2) The Swin Transformer Block of each layer performs feature mapping-based knowledge distillation on the corresponding ConvNeXt Block, respectively. (3) The softmax output layer of the Swin Transformer performs knowledge distillation based on logits output to ConvNeXt.

**FIGURE 5:**
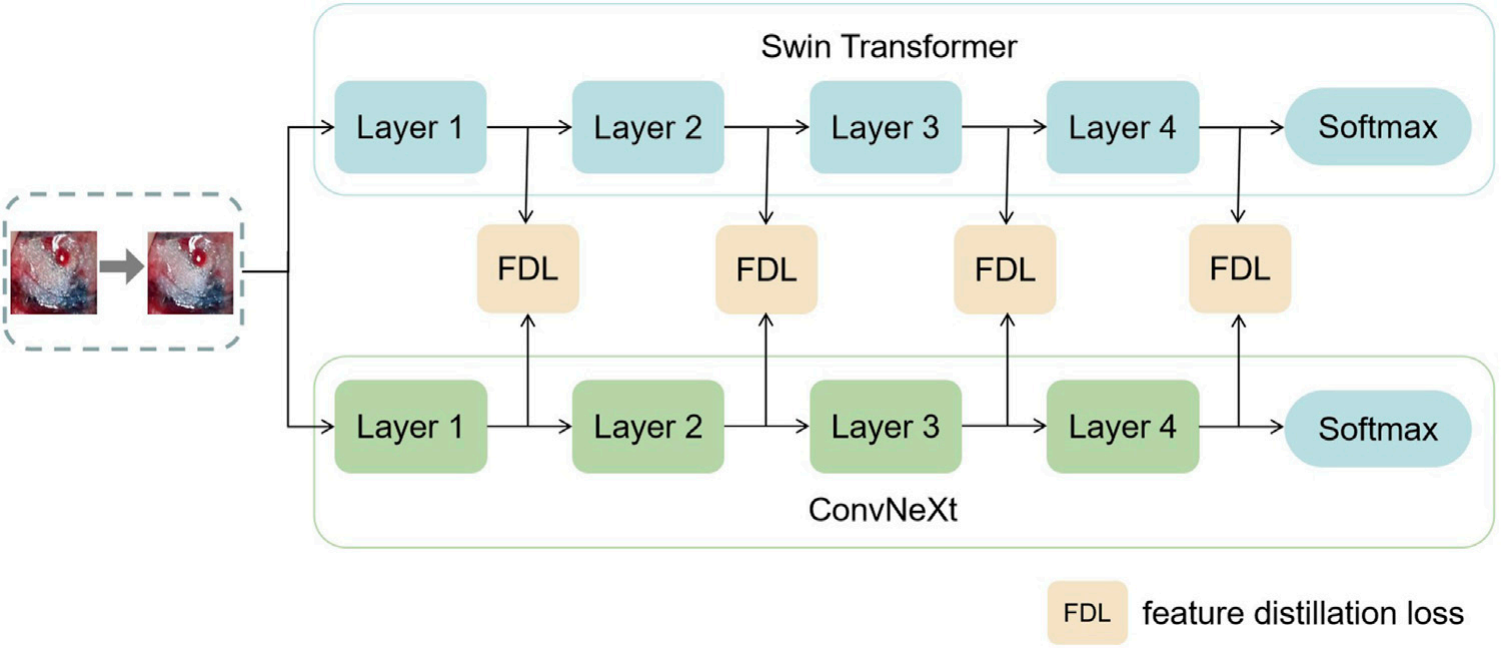
This figure shows the specific process of feature distillation. (1) Firstly, the datas are synchronously input into the teacher model and student model during the training process. (2) The feature maps are obtained from each intermediate network layer of the teacher model and the student model. (3) The feature maps from the teacher model and the student model are transformed to the same dimension and then use the absolute value to measure the similarity between the knowledge. (4) We calculate the distillation loss function of the intermediate layer and optimize the student using the backpropagation algorithm Neural Network.

**FIGURE 6:**
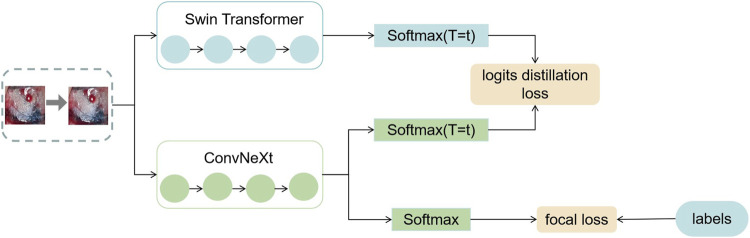
This figure shows the exact process of logits distillation. (1) We train the teacher model Swin Transformer and use the distillation temperature (T) to generate soft labels. (2) This work uses the soft labels and the distillation temperature (T) as well as the hard labels to train the student model ConvNeXt at the same time. (3) We compute the corresponding distillation loss and then compute the overall student model’s loss function and optimize the student model using the backpropagation algorithm.

In this section, this work proposed the distillation module as shown in Figure 4. We constructed the distillation framework along the following lines: first, the ConvNeXt and Swin Transformer were each divided into several corresponding modules based on their original structures (Zhang et al., 2019). During the training period, the Swin Transformer was utilized as the teacher model, and the Swin Transformer Blocks were converted to the matching ConvNeXt Blocks for feature mapping, respectively (Romero et al., 2014). To match the feature mapping output of the Swin Transformer Blocks, a regressor was introduced after the ConvNeXt Blocks for the feature boosting. To suit the feature mapping of the Swin Transformer’s visual field information, the knowledge from the Swin Transformer’s feature mapping was incorporated into ConvNeXt at each layer by computing the L2 loss of the Swin Transformer Blocks’ feature output with the regressor modified to induce its feature mapping on each of its block layers, illustrated by Figure 5. In addition, the softmax layers from the Swin transformer supervised the ConvNeXt’s output, and the Swin Transformer was utilized to provide soft goals as supervision to transfer information from the teacher model to the student model (Hinton et al., 2015). The relevance of each soft target was managed by introducing a temperature factor termed T (Hinton et al., 2015). The output of the softmax probability distribution tended to be smoother as T increased, as shown in Figure 6. The information carried by the categories with fewer samples would be comparatively enhanced, and the training of the student network would concentrate more on the type with fewer samples.

The specific training process was therefore completed in three stages. In the first stage, knowledge was transferred from the teacher network to the student network using an adaptive layer (1 × 1 convolutional layer) to cause the feature mapping of each layer of ConvNeXt to adapt the feature mapping of Swin Transformer’s visual field information on a similar feature space. In the second stage, the student network was trained under the supervision of the soft labels supplied by the teacher network, which meant that the student network’s training relied on the contribution of the teacher network to identify categories with small sample sizes. The difference between the predicted logits produced by the ConvNeXt and the accurate labels was computed in the third stage using the focal loss function (Lin et al., 2017). This clinical dataset’s category data were drastically out of balance. Thus, a weighting factor was introduced to the loss function compared to the widely-used cross-entropy function to give a few categories more weight and balance the loss function’s distribution (Loshchilov and Hutter, 2018).

### 3.3 Loss function design

In order to improve the performance of the model, three loss functions were introduced during the training process:

• Matching L2 loss between feature maps: it was obtained by calculating the L2 loss between the feature mapping of Swin Transformer Blocks and ConvNeXt Blocks. The knowledge from the feature mapping extracted by the teacher network was introduced into the student network through the L2 loss so that the feature mapping of the ConvNeXt Block matches the feature mapping of the Swin Transformer Blocks:

$${Loss}_{1}={\left\|\phi_{t}\left(f_{t}\left(x\right)\right),\phi_{s}\left(f_{s}\left(x\right)\right)\right\|}_{2}^{2}$$ (1)

where *f*_*t*_(*x*) and *f*_*s*_(*x*) are the middle layer feature maps of the teacher model and the student model, respectively. The conversion function $\phi_{t}\left(f_{t}\left(x\right)\right)$ and $\phi_{s}\left(f_{s}\left(x\right)\right)$ is usually applied when the feature maps of the teacher and student models are not of the same shape and represents the similarity function that matches the feature maps of the teacher and student models.

• Matching logit output between the teacher model and the student model: the teacher network provided soft labels to induce the training of the student network, giving the student network better generalization capabilities. The importance of each soft target is controlled by introducing a temperature factor T:

$$p_{i}=\frac{\exp\left(z_{i}/T\right)}{\sum_{j}\exp\left(z_{j}/T\right)}$$ (2)

where *Z*_*i*,*j*_ is the output of the softmax layer after the fully connected layer. T typically denotes the distillation temperature, with higher temperatures producing weaker probability distributions over the classes. Distillation loss is calculated using the output of the softmax layer between the student and teacher networks as follows:

$${Loss}_{2}=\underset{i}{\sum }-p_{i}\left(z_{ti},T\right)\log\left(p_{i}\left(z_{si},T\right)\right)$$ (3)

where *z*_*t*_ and *z*_*s*_ are the logits outputs of the teacher and student models respectively, and the student model matches the logits output of the teacher model by the cross-entropy gradient.

• The focal loss from the labels to the student network: it is calculated using the labels of the clinical dataset and the output of the softmax layer of the student network:

$${Loss}_{3}=\overset{5}{\underset{t=1}{\sum }}-\alpha_{t}{\left(1-p_{t}\right)}^{\gamma }\log\left(p_{t}\right)$$ (4)

where *α*_*t*_ denotes the weight assigned to each category, and *p*_*t*_ indicates the probability that each sample is predicted to be a specific category, reflecting the proximity to the ground truth. The larger *p*_*t*_ is the more accurate the classification is. Therefore, the focal loss function is equivalent to increasing the weight of the category with a smaller number of samples in the loss function, making the loss function more inclined to the category with a smaller number of samples.

In summary, the total loss function used in this experiment is defined as follows:

$$\text{ Loss }=\alpha Loss1+\left(1-\alpha \right)Loss2+\beta Loss3$$ (5)

Here, *α* and *β* are the weighting coefficients used to balance the three loss functions. We tested various values of *α* and *β* in the training phase and analyzed the variation curve of the total loss function when *α* and *β* take different values, and finally, we chose *α* = 0.5 and *β* = 1 as the final weighting coefficients used in this thesis.

## 4 Experiments

### 4.1 Hardware and software platform environment

The experiments are based on the Pytorch framework platform to implement the KD-ConvNeXt model, using the OpenCV-python library as an image preprocessing tool. The processor of the computing platform on which the experiments are conducted is Intel(R) Xeon(R) CPU E5-2,678 v3 @ 2.50GHz, the memory is 236G, and the graphics card model is NVIDIA Corporation TU102 [GeForce RTX 2080 Ti] with 12 GB of video memory.

### 4.2 Dataset processing

The dataset used in this paper is a lung tissue photograph taken with available camera equipment. It presents mainly some morphological features in the natural state, and its normality, resolution, and other indicators are weak. In addition, since the clinical image dataset taken in the field undergoes various processes that make the images blurred and degraded, such as camera blurring and image compression when cropping the ROI region, the Real-ESRGAN algorithm (Wang et al., 2021) was used to process the image dataset to improve the resolution of the images to remove the noise. All lesion data were divided into training sets, validation sets, and test sets according to the ratio of 8:1:1, where 1778 images were in the training sets, and there were 234 and 209 images in the validation and test sets, respectively. Since the model requires an input size of 224 × 224 × 1 for the images, the extracted lung tumor images were resized to the same size before being inputted into our model. Details of the data distribution of the five subtypes of lung adenocarcinoma and the specific classification table are shown in Table 1.

**TABLE 1:** Details of the number of categories in the five categories of the clinical data set and the division of the sample.

Pathological subtypes of lung adenocarcinoma	Total number	Train sets	Val sets	Test sets
Grade 1	41	33	3	5
Grade 2	1,317	1,054	141	122
Grade 3	196	157	23	16
AIS	121	97	12	12
MIA	546	437	55	54

The training of the model requires a sufficient amount of training data available, which may lead to overfitting if only a small amount of training data is used. To prevent overfitting due to the limited number of images and to maximize the generalization performance of the model, 1778 images in the training dataset are augmented. For example, random horizontal and vertical flipping, random cropping of images (cropping rate up to 10% of the original image), random translation (10% in the *x*, *y*-axis direction), etc., followed by normalization of images, improved the robustness and generalization ability of the model.

### 4.3 Experimental implementation details

The algorithmic model proposed in this paper is constructed by Pytorch, and the convolution kernel is set to the initial value setting method proposed by (Liu et al., 2022). The pre-training of the layers is performed by stochastic gradient descent with a learning rate of 0.001 using the AdamW optimizer. Since the pre-training weights outperformed the random initialization weights, the initial learning rate was set to 0.001, the momentum was initialized to 0.9, the weight decay parameter was initialized to 5e-4, the epoch was set to 100, and the training batch size was set to 32. We initialize the model parameters to the weights saved after pre-training to improve the training process and performance. Specifically, the model’s pre-trained weights other than the head are frozen. The softmax layer output of the model predicts the probability of belonging to a category for each image in the test set, and the category with the highest probability is selected as the category for the prediction output. To ensure that the model performance reported on the test set is not due to accidental training validation test set partitioning, the training validation test set partitioning and model training process is repeated five times. Each time we retrained the model and re-optimized all parameters from scratch to ensure the robustness of the results.

### 4.4 Experimental evaluation indexes

To assess the performance of the model on the clinical dataset, the following metrics are measured (where precision and recall are measured by considering a category as a positive category and the rest as negative categories when considering a category):

• Accuracy: the number of correctly classified samples as a percentage of all samples and is used to measure the percentage of correctly classified images. However, no distinction is made between the different categories, so the error rate and accuracy under specific categories are not known.
• Precision: the ratio of the number of correctly classified positive samples to the number of all predicted positive samples of the classifier.
• Recall: the ratio of the number of correctly classified positive samples to the number of actual positive samples, also known as sensitivity or true positive rate.
• F1-score: a weighted average of precision and recall to balance precision and recall. For uneven class distribution, the F1-score is more useful for evaluating models.

$$\text{ Accuracy }=\frac{TN+TP}{FP+TN+TP+FN}$$ (6)

$$\text{ Precision }=\frac{TP}{TP+FP}$$ (7)

$$\text{ Recall }=\frac{TP}{TP+FN}$$ (8)

$$\text{F}1-\text{score}=\frac{2*\text{ precision }*\text{ recall }}{\text{ precision }+\text{ recall }}$$ (9)

Where TP denotes the number of positive samples classified as positive samples, FN denotes the number of positive samples labeled and classified as negative samples, FP denotes the number of negative samples labeled and classified as positive samples, and TN denotes the number of negative samples labeled and classified as negative samples. In calculating the precision, the recall and F1 score values of the whole confusion matrix, the precision degree, recall, and F1 score values of each category are calculated first and then averaged.

## 5 Analysis of experimental results

### 5.1 Comparative experimental results and analysis

The evaluation metrics defined by the above equations were used as quantitative evaluation metrics to evaluate the model’s effectiveness for lung cancer image classification. This work chose ConvNeXt and Swin Transformer as the baseline models for the experiments and three advanced network structures such as EfficientNet V2 (Tan and Le, 2021), DaViT (Ding et al., 2022) and CoAtNet (Dai et al., 2021) as the comparative experimental models. This work trained each model under the same experimental conditions (loss function, learning rate, optimizer, etc.). This work saved the model that performed best on the validation set and evaluated it on the test set. Finally, the experimental results showed that the model proposed in this paper had a better classification performance and outperforms other comparative models on the test set.

**FIGURE 7:**
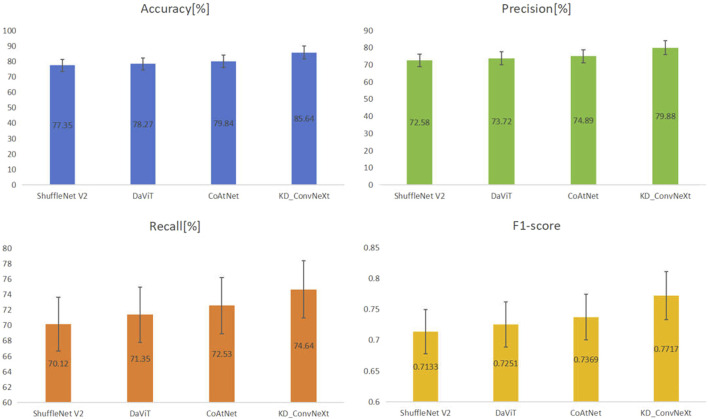
Results of comparing KD_ConvNeXt with other comparative models on the test set (bolded font indicates optimal values).

The results of the comparison experiments are shown in Figure 7, which shows the performance of each model on the test set. This work used four evaluation metrics to measure the performance of the classification results, namely accuracy, precision, recall, and F1-score. This work predicted and calculated the evaluation metrics for each test sample, and finally obtained the average of the evaluation metrics of the models on the test set. This work shows these results in the figure, from which it can be seen that our method achieved better results than other models for lung cancer classification on the clinical test set. Among all the compared models, CoAtNet performed better, with an accuracy of 79.84%, respectively. Our method still outperformed all comparative models, with accuracy reaching the highest level of 85.64%, which basically met the clinical level requirement. The above experimental results validate the superiority of our proposed method compared with other methods.

### 5.2 Analysis of ablation experiments

In this section, we used ablation experimental analysis to demonstrate the validity of the knowledge provided by the teacher network in the model. This work chose ConvNeXt as the baseline model for the experiments. We used the Swin Transformer as the teacher model to provide knowledge distillation based on logit output and feature-based, respectively. Table 2 compares the performance of the ablation experimental models on the test set. Logits-based and features-based distillation significantly improves the classification performance of ConvNeXt and allows it to converge to lower loss values during training. This is because the logits-based distillation approach provides the student network with soft labels to supervise the student network’s training, making the student network’s training dependent on the teacher network’s contribution to identifying classes with small sample sizes. The features-based distillation approach, on the other hand, improves the ability of the student network to fit features based on the similar structural features of ConvNeXt and Swin Transformer. The ablation experiments demonstrate the effectiveness of our proposed distillation approach. It can be found that the final proposed KD ConvNeXt is the best among all the selected evaluation metrics. Also, our proposed modules, such as logits and feature distillation, improve the performance of ConvNeXt to some extent.

**TABLE 2:** Results of KD ConvNeXt ablation experiments on the test set (bolded font indicates optimal values).

Method	Accuracy [%]	Precision [%]	Recall [%]	F1-score
ConvNeXt	81.12	67.34	68.50	0.6791
Swin Transformer	82.75	70.48	71.40	0.7093
ConvNeXt + logits distillation	83.05	72.38	72.54	0.7246
ConvNeXt + feature distillation	84.21	74.20	74.46	0.7433
KD_ConvNeXt	**85.64**	**79.88**	**74.64**	**0.7717**

The bold values indicate the optimal value.

### 5.3 Confusion matrix analysis of the model

**FIGURE 8:**
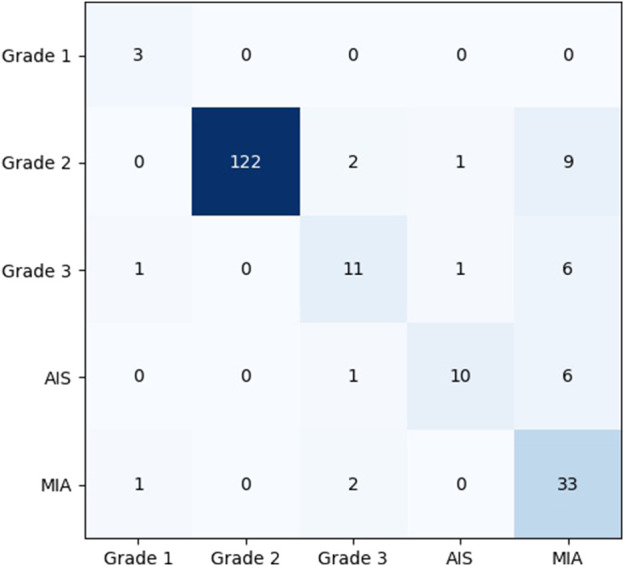
Confusion matrix analysis of the model on the test set.

To show the effects and problems of the model in practical applications more clearly, the confusion matrix of the model on the test set can be analyzed and discussed. Figure 8 shows the classification confusion matrix of KD_ConvNeXt on the test set of clinical data, where the horizontal coordinates indicate the true label categories and the vertical coordinates indicate the predicted label categories. The diagonal line represents the number of the model’s predictions and labels that agree with each other: the larger the number on the diagonal line, the better it represents the model’s prediction results in that class. As shown in the figure, for the Grade 1 category images, 5 images were used for testing. In total, 3 of the images were correctly predicted, which is a better prediction result for Grade 1 with a small number of samples. The Grade 2 category images were tested using 122 images, and all predictions were correct, with a slightly higher discriminatory ability compared to the other categories, probably because the Grade 2 accounts for most of the data in the clinical training data set, and the model fits the features of the Grade 2 images very well. In the Grade 3 and AIS categories, the number of categories incorrectly predicted to be classified as other categories was low. However, 9 images were incorrectly classified as Grade 2 for the MIA category, and another 6 were incorrectly predicted as Grade 3 and AIS, respectively. The results indicate that our model can predict the pathological type of lung tumors. However, the accuracy of lung tumor prediction classification is still needed to improve to assist clinical treatment, for example, to reduce the recall of MIA category prediction. Table 3 shows the precision and recall of the proposed method for each lung tumor category.

**TABLE 3:** Precision and Recall of each category predicted by KD_ConvNeXt on the test set.

Type	Precision [%]	Recall [%]
Grade 1	1	0.6
Grade 2	0.9104	1
Grade 3	0.5789	0.6875
AIS	0.5882	0.8333
MIA	0.9167	0.6111

## 6 Conclusion

This paper established a clinical dataset of section images of lung tumor surgical specimens. We proposed a classification model based on logit output distillation and feature distillation to solve the pathological classification of lung tumors. Our method tested the method on the dataset, calculated classification evaluation metrics, and compared several advanced image classification methods. The results showed that our method achieved the best results on each metric. The proposed approach also designed ablation experiments to demonstrate the effectiveness of the proposed knowledge distillation module and analyze its effectiveness. The results of the ablation experiments showed that each of our proposed distillation modules can improve the performance of ConvNext to some extent. Our method and technical route are better able to assist the surgeons in deciding on subsequent surgical steps and treatment strategies than intraoperative frozen pathology analysis that requires at least half an hour or more. However, the model still needs to be improved in terms of its effectiveness in addressing the long-tail effect and needs to be supported by a larger clinical dataset before it can be clinically applied, and the classification accuracy effect needs to be improved to match the intraoperative freezing results. We plan to conduct more extensive experiments in the future using the large number of samples provided by Guangdong Provincial People’s Hospital to solve the problem of unbalanced sample size. On the other hand, we plan to further optimize and lighten the whole model in the follow-up work to reduce the number of model parameters without losing accuracy to better assist in the automatic, rapid, accurate, and efficient classification of lung cancer.

## Data Availability

The datasets presented in this article are not readily available because No. Requests to access the datasets should be directed to 2021094681@qq.com.
