# Supplementary material for: KD_ConvNeXt: knowledge distillation-based image classification of lung tumor surgical specimen sections
Source: Front Genet. 2023 Sep 18;14:1254435. doi: 10.3389/fgene.2023.1254435 (PMC10544998; doi:10.3389/fgene.2023.1254435)
Supplement: Supplementary file 2 [file Presentation1.pdf]

The DOI for

1. Anderson and Davis, 2018: 10.1001/jama.2018.16336
2. Camalan et al., 2021: 10.3390/cancers13061291
3. Cho et al., 2019: 10.1055/a-0981-6133
4. Dai et al., 2021: 10.48550/arXiv.2106.04803
5. Esteva et al., 2017: 10.1038/nature21056
6. Halder and Dey, 2023: 10.1016/j.bspc.2023.105149
7. Han et al., 2021: 10.1007/s00259-020-04771-5
8. Hu et al., 2019: 10.1093/jnci/djy225
9. Masud et al., 2021: 10.3390/s21030748
10. Shkolyar et al., 2019: 10.1016/j.eururo.2019.08.032
11. Sun et al., 2020: 10.1007/s00330-020-06776-y
12. Viale,2020: 10.6004/jadpro.2020.11.2.1
13. Xu et al., 2023: 10.1007/s10489-022-03486-4
14. Yang et al., 2017: 10.1016/j.media.2017.08.006
15. Zhang et al., 2022:
16. Zhang et al., 2023: doi:10.1109/TPAMI.2022.3200344
