# Supplementary material for: KD_ConvNeXt: knowledge distillation-based image classification of lung tumor surgical specimen sections
Source: Front Genet. 2023 Sep 18;14:1254435. doi: 10.3389/fgene.2023.1254435 (PMC10544998; doi:10.3389/fgene.2023.1254435)
Supplement: Supplementary file 3 [file Presentation3.zip › Proof/Q9.docx]

\begin{abstract}

\section{}

\textbf{Introduction:} Lung cancer is currently among the most prevalent and lethal cancers in the world in terms of incidence and fatality rates. In clinical practice, identifying the specific subtypes of lung cancer is essential in diagnosing and treating lung lesions.\\

\textbf{Methods:} This paper aims to collect histopathological section images of lung tumor surgical specimens to construct a clinical dataset for researching and addressing the classification problem of specific subtypes of lung tumors. Our method proposes a teacher-student network architecture based on a knowledge distillation mechanism for the specific subtype classification of lung tumor histopathological section images to assist clinical applications, namely KD\_ConvNeXt. The proposed approach enables the student network (ConvNeXt) to extract knowledge from the intermediate feature layers of the teacher network (Swin Transformer), improving the feature extraction and fitting capabilities of ConvNeXt. Meanwhile, Swin Transformer provides soft labels containing information about the distribution of images in various categories, making the model focused more on the information carried by types with smaller sample sizes while training.\\

\textbf{Results:} This work has designed many experiments on a clinical lung tumor image dataset, and the KD\_ConvNeXt achieved a superior classification accuracy of 85.64\% and an F1-score of 0.7717 compared with other advanced image classification methods.

\end{abstract}
