# Supplementary figures and images for: KD_ConvNeXt: knowledge distillation-based image classification of lung tumor surgical specimen sections
Source: Front Genet. 2023 Sep 18;14:1254435. doi: 10.3389/fgene.2023.1254435 (PMC10544998; doi:10.3389/fgene.2023.1254435)

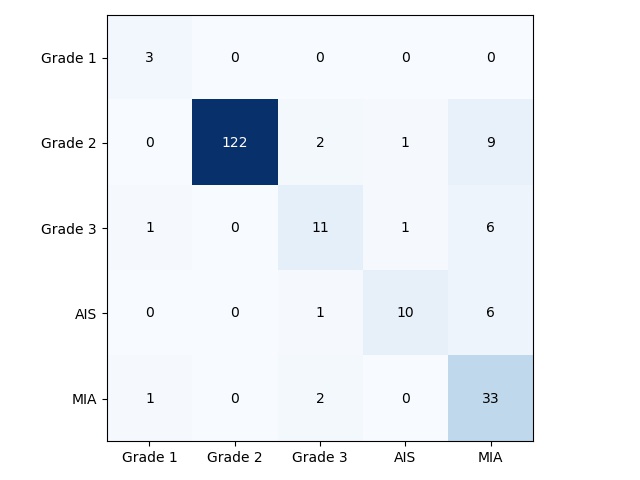

Supplement: Supplementary file 3 [file Presentation3.zip › Proof/retu.jpg]
